# Generative Deep Learning for de Novo Drug Design—A Chemical Space Odyssey

**DOI:** 10.1021/acs.jcim.5c00641

**Published:** 2025-07-09

**Authors:** Rıza Özçelik, Helena Brinkmann, Emanuele Criscuolo, Francesca Grisoni

**Affiliations:** Department of Biomedical Engineering, Institute for Complex Molecular System (ICMS) and Eindhoven AI Systems Institute (EAISI), 3169 Eindhoven University of Technology, Eindhoven 5612 AZ, Netherlands

## Abstract

In recent years, generative deep learning has emerged as a transformative approach in drug design, promising to explore the vast chemical space and generate novel molecules with desired biological properties. This perspective examines the challenges and opportunities of applying generative models to drug discovery, focusing on the intricate tasks related to small molecule generation, evaluation, and prioritization. Central to this process is navigating conflicting information from diverse sources—balancing chemical diversity, synthesizability, and bioactivity. We discuss the current state of generative methods, their optimization, and the critical need for robust evaluation protocols. By mapping this evolving landscape, we outline key building blocks, inherent dilemmas, and future directions in the journey to fully harness generative deep learning in the “chemical odyssey” of drug design.

## Graphical abstract

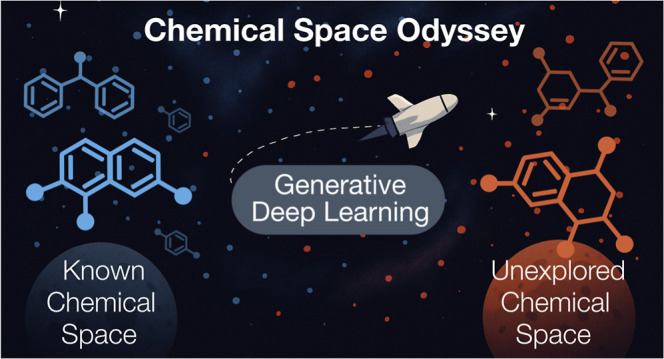

## 1 Introduction

Generative deep learning has emerged as a transformative tool in de novo drug design, and in the molecular sciences at large.^1–3^ By leveraging existing molecular data, generative deep learning enables the on-demand generation of molecules that possess desirable properties.^4^ With the “chemical universe” estimated to contain up to 10^60^ drug-like molecules,^5^ generative deep learning has accelerated the discovery of novel compounds, compared to traditional rule-based molecular assembly and enumeration approaches.^6–10^ In less than a decade since its introduction in drug discovery,^11^ generative deep learning has been extensively applied in prospective wet-lab studies^12–19^ and demonstrated its potential in real-world applications. Meanwhile, the development of approaches to better explore the chemical universe is gaining momentum in the cheminformatics community.^20–23^ Currently, we are observing a productive synergy between computer science advances and insights rooted in chemical and biological knowledge.

While generative deep learning is making great strides in designing promising molecules, the understanding of what model to choose and how to prioritize molecular candidates remains limited. With an ever-growing pool of methods available for molecular generation and optimization, choosing the most suited approach might not always be straightforward. Despite the availability of benchmarks,^10,24^ retrospective comparisons are poised to be incomplete for new, prospective applications. Researchers might face many “traps”, such as overfitting to specific datasets or overlooking key molecular properties.^25,26^ Moreover, the generated designs can exhibit limited synthesizability,^27^ and current synthetic accessibility scores might struggle to capture the effect of subtle structural variations, reaction selectivity, and the availability of building blocks essential to chemical reactions.^28^ Finally, optimizing conflicting properties—such as balancing pharmacophore similarity with structural diversity—presents another layer of complexity. As a result, de novo molecule design through generative methods continues to be an odyssey (from Greek “Oδúσσεια”, meaning “the journey of Odysseus” or “a long and adventurous journey”) in chemical space.

This perspective highlights open questions, “known unknowns”, and key challenges in generative small molecule design, with a particular focus on ligand-based approaches. After reviewing current generative approaches for de novo design, we offer insights and propose potential future directions. By highlighting both the caveats and advantages of these methods, we venture to forecast the exciting possibilities that lie ahead.
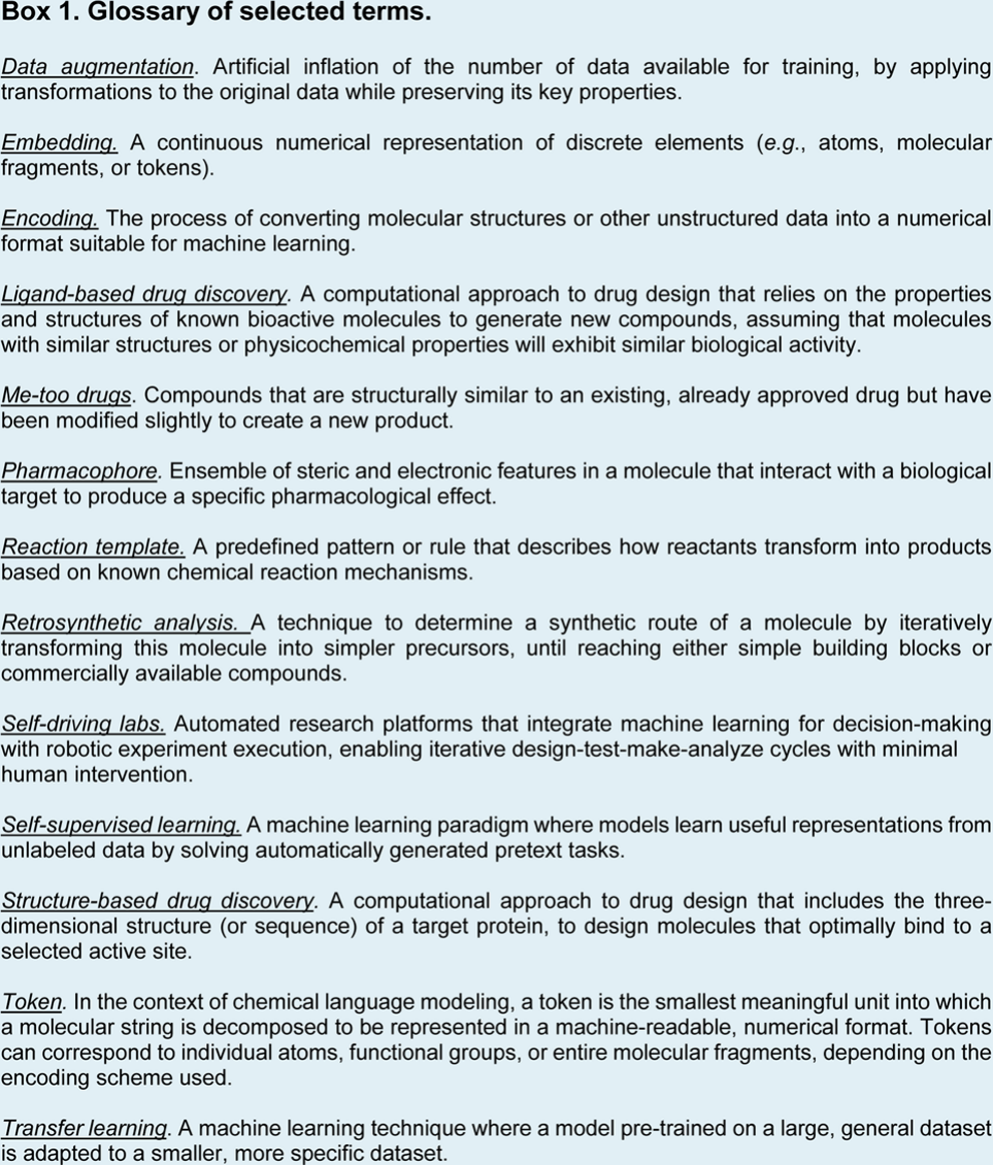


## 2 Charting the Chemical Cosmos with Generative AI

### 2.1 Molecular Representations for Deep Learning

How to represent molecular information has been a central scientific question for over a century.^29–32^ Molecular representations are “a symbolic transformation of [chemical] reality”,^33^ initially devised for the purpose of communication and human reasoning.^33^ Examples are the ball-and-stick model, Kekulé’s representation,^34^ and Lewis structure of molecules.^35^ Such models of chemical reality were progressively developed to address the complexity of molecular structures at different levels of abstractions. From the mid of the 20th century, with the advent of computers and the need for machine-readable formats, string-based molecular representations (notations) have started to be developed, such as Wiswesser Line Notation^36^ and the SMILES (Simplified Molecular Input Line Entry System)^37^ strings. These representations were initially proposed to complement systematic chemical nomenclature,^36^ and enabled the encoding of chemical structures in a compact format suitable for information storage, processing, and retrieval.

With the introduction of machine learning to cheminformatics, the role of molecular representations has also evolved.^38^ Molecular representations have become a fundamental step to encode chemical information for machine learning and data analysis. Their role has further become more prominent since the advent of deep learning.^39^ Certain deep learning algorithms can handle non-structured (i.e., non-tabular) input, allowing the direct usage of some of these molecular representations, without the need of feature engineering. This shift enables generative deep learning to use some molecular representations as inputs to generate novel chemical structures in an end-to-end fashion.^38,40^ In other words, models can be trained to directly learn from and generate molecules in the form of a chosen molecular representation. To date, the following molecular representations have played a key role in the field of generative deep learning for de novo design:

- *Molecular strings.* Molecular strings represent a molecule as a sequence of characters. The most popular string notation to date is the SMILES notation.^37^ SMILES strings are obtained by traversing a two-dimensional molecular representation (molecular graph), and annotating the atomic symbols and bond types along the traversal path. Rings and branches are encoded using numeric labels and parentheses, respectively, to capture molecular connectivity while maintaining a linear format (Figure 1b). SMILES can be seen as a chemical language,^37^ possessing its own syntax rules to map this string representation back to a chemically valid molecule. Since the early successes of SMILES strings as a representation for de novo design,^11,12^ several extensions to this notation have been developed. DeepSMILES^41^ (Table 1) were proposed to address aspects that often caused invalidity of the generated SMILES (brackets and ring characters), but have found limited application, due to their difficult syntactic rules.^42^ The Self-referencing embedded strings (SELFIES)^43^ are built upon “semantically constrained graphs”, in such a way that SELFIES strings always corresponds to a valid molecule (Table 1). While SMILES and SELFIES achieve a similar performance overall, they come with distinct advantages and drawbacks. The enforced validity of SELFIES might be ideal to generate complex macromolecular entities, whose structural complexity increases the likelihood of generating invalid SMILES (e.g., natural products^20,44^ ). SMILES strings might be more ideal for distribution learning, to as the generation of invalid SMILES allows to filter out low-quality molecular designs, which would not be possible when validity is enforced like in the SELFIES language.^45^ Recently, great attention has been given to string representations that are based on molecular fragments, such as Sequential Attachment-based Fragment Embedding (SAFE),^46^ GroupSELFIES^47^ and fragSMILES.^48^ These representations have been developed to provide “chemically-rich” molecular representations, to better learn the properties of the training molecules, and/or provide a better representation of molecular chirality (fragSMILES,^48^ Table 1).
- *Two- and three-dimensional molecular graphs.* Graphs are among the most intuitive ways to represent molecular structures (Figure 1c). Any molecule can be represented as a mathematical graph *G* = (*V*, *E*), whose vertices (*v*_*i*_ ∈ *V*) represent atoms, and edges (*e*_*ij*_ ∈ *E*) constitute their connections (bonds). Additionally, features of atoms and/or edges can be added to further characterize the graph.^49^ Two-dimensional (2D) molecular graphs usually consider only topological and chemical features (e.g., bond and atom types). Three-dimensional information (3D) can also be added to the features of the molecular graphs, such as torsional angles, or three-dimensional coordinates.^38^ Generating molecules in the form of graphs has attracted a lot of attention, for both ligand- and structure-based drug discovery.^50–54^ A particularly recent direction in the field is the attempt to generate 3D molecular graphs,^21,55–57^ in the attempt to better capture properties that depend on the spatial arrangement of atoms (e.g., protein binding). Moreover, 3D graphs can also be encoded as the so-called point clouds,^21,22,58^ i.e., by omitting bond information and encoding the spatial positioning of atoms (absolute or relative coordinates). Once the geometry is generated in the form of point cloud, bond type can be constructed by dedicated chemistry software (e.g., Open Babel^59^).
- *Molecular surfaces.* In addition to 3D graphs and point clouds mentioned above, a limited number of studies have represented molecules via their molecular surface, which encloses the 3D structure of a molecule at a certain distance from each atom (Figure 1d). Surfaces are then usually represented as (a) 3D meshes^60,61^ (a set of polygons describing the coordinates in the 3D space), (b) 3D point clouds,^62,63^ that is, a collection of discrete points in 3D space that define the surface geometry without explicit connectivity between them, or (c) 3D voxels,^64,65^ that is three-dimensional grid of cubic cells where each voxel represents a volume element in space, allowing for the discretization of the surface and surrounding molecular structure into a regular grid format. Surfaces can then be further characterized by additional chemical (for example, hydrophobic, electrostatic) and geometric (such as local shape, curvature) features. While surface representations have mostly gathered popularity to capture information on protein surfaces or cavities, and protein–protein interactions,^60,65^ they have also been used to generate molecules matching desired ligand shapes,^66,67^ or conditioned on the information on surface features.^68^

**Table 1:** Examples of Molecular String Notations, for the Molecule Caffeine (Figure 1a). SMILES and canonical SMILES differ based on the atom ordering used to write the SMILES string (deterministic when canonicalization is applied).

Notation	String
InChi	1S/C8H10N4O2/c1-10-4-9-6-5(10)7(13)12(3)8(14)11(6)2/h4H,1-3H3
Canonical SMILES^37^	Cn1cnc2c1c(=O)n(c(=O)n2C)C
SMILES^37^	CN1C=NC2=C1C(N(C(N2C)=O)C)=O
DeepSMILES^50^	CN(C(=O)N(C(=O)C=NC)C)C
SELFIES^43^	[C][=N][C][=C][C][Branch1_1][C][=O][C][Branch1_1][C][=O][C][Branch1_1][C][Ring1][Branch1_1][C][Ring1][Branch1_1][C]
fragSMILES^44^	C.<2>O=c1[nH]c(=O)c2[nH]cnc2[nH]1<6>.<10>(C.).C.

Once a molecular representation has been chosen, a fundamental step is molecule encoding. Encoding is the transformation of molecular structures into numerical representations that can be processed by machine learning models. Encoding ensures that the chosen molecular representation is converted into a format suitable for deep learning without loss of information. For molecular strings (Figure 1e) common methods include one-hot encoding, which converts each token into a unique binary vector, and learnable embeddings, which represents tokens as vectors of unique numbers that are updated during training. For molecular graphs (Figure 1f), encoding involves constructing^49^ an adjacency matrix that defines atomic connectivity and a node features matrix to describe atomic properties. Optionally, edge features can also be included to specify bond characteristics (e.g., bond type or order). Recent studies on bioactivity prediction show that molecular strings encoding strategy has little impact on the model performance,^69^ whereas the choice of edge features is crucial and task-specific.^49^

**Figure 1:**
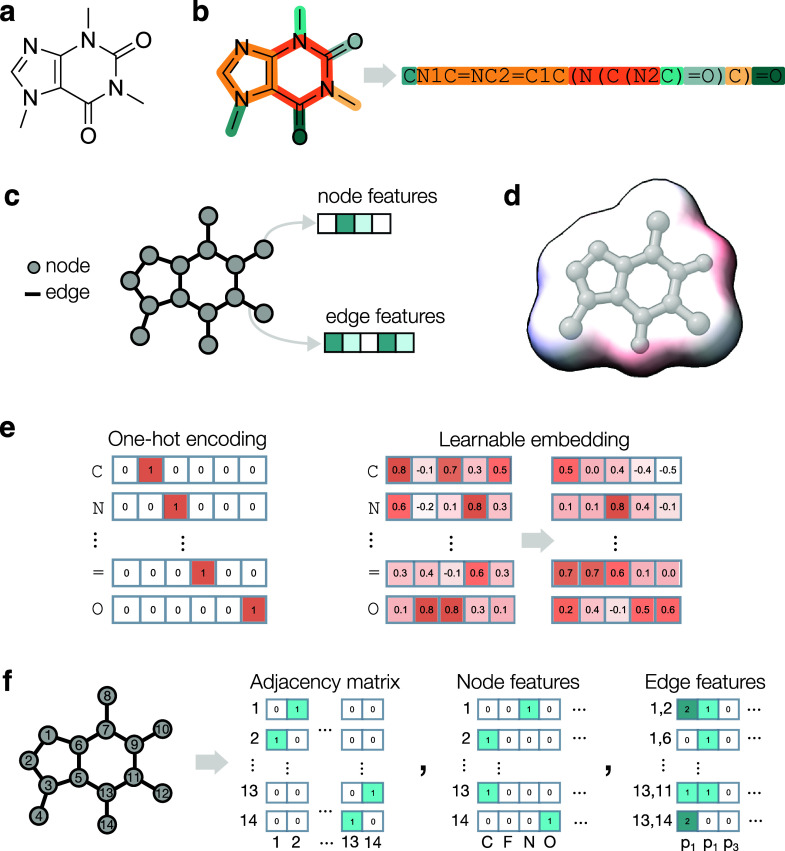
**Representing molecular information for deep learning.**(a) Selected molecular example (caffeine). (b–d) Commonly used “raw” molecular representations. (b) Simplified Input Line Entry Systems (SMILES) strings, which capture two-dimensional molecular information as a set of textual characters (“tokens”). (c) Molecular graph, whose vertices represent atoms, and edges constitute their connections (bonds). Additionally, features of atoms and/or edges can be added to further characterize the graph, such as chemical and bond properties, as well as three-dimensional information. (d) Molecular surface, which encloses the 3D structure of a molecule at a certain distance from each atom. (e,f) Encoding of a molecular representation into a “machine readable” format. (e) Encoding of molecular strings (SMILES strings, in the selected example). One-hot encoding represents each token with a unique binary vector. Learnable embeddings start from a random vector per token and are updated during training to improve the model performance. (f) Encoding of molecular graphs. The adjacency matrix captures information about atoms bound to each other (i.e., connected by one edge). Node features can be specified for each atom (e.g., atom type, in this example). Features for edges can also be specified (in this example: p_1_ = bond order, p_2_ = ring membership, p_3_ = aromaticity).

Every molecular representation (and corresponding encoding) requires ad hoc neural network architectures to learn and aggregate molecular information,^50^ and shows distinct advantages and disadvantages. To date, models based on molecular string representations (often referred to as chemical language models) are the ones that have found extensive experimental validation to generate bioactive molecules (see Section 3.4; Table 4).^12,13,15,17,19,70^ Early studies have demonstrated that relying on string representations for de novo design better captures complex molecular properties compared to molecular graphs.^71^ Nonetheless, there is ample room for improvement when it comes to representing and generating molecules de novo, since molecular strings capture only 2D information (molecular topology and composition). In this context, methods that learn sophisticated three-dimensional information (e.g., geometry and chemistry of binding pockets or known molecular conformers) to generate molecules matching relevant binding motifs^72–76^ is a particularly enticing direction. However, to date, it might be difficult for deep learning models to generate molecules with valid 3D conformations, for instance, in terms of torsional angles and bond lengths,^77,78^ albeit current advances show great promise to overcome such issues.^79–81^

### 2.2 Deep Learning Architectures for Drug Discovery

A plethora of generative deep learning architectures was proposed for designing small molecules with desired properties (e.g., bioactivity toward a pharmacologically relevant target). In its simplest form, generative drug discovery can be cast as a ligand-based task,^11,82,83^ where models learn from the molecular structures of bioactive molecules and generate novel compounds with desirable characteristics, without requiring explicit knowledge of the target protein’s structure or sequence. Structure-based approaches also exist, which leverage structural (or sequence) information about the target protein to guide molecular design.^51,54^ For simplicity, in this perspective, we focus mostly on ligand-based approaches, and strategies for structure-based de novo design are discussed elsewhere.^51,54^ Regardless of whether ligand- or structure-based strategies are employed, the effectiveness of generative models hinges on how molecular information is encoded and processed by deep learning approaches for follow-up molecular generation. In general, different molecular representations require tailored neural architectures to suitably process and leverage the molecular information they encode.

#### 2.2.1 Molecular Strings

One of the earliest and most widely adopted approaches in ligand-based drug design is chemical language modeling (CLM).^4,9,11^ CLMs take inspiration from natural language processing and treat molecule design as a sequence generation problem. CLMs are trained to predict the next token in a molecular string given the preceding ones. This self-supervised learning approach enables models to generate valid molecular sequences while capturing underlying chemical patterns.^71^ Moreover, CLMs have often been applied in combination with “data-augmentation”, where multiple SMILES (or SELFIES) strings are used to represent the same molecule. This is usually achieved by enumeration, where multiple molecular strings—obtained by traversing the molecular graph in different directions or starting from different atoms—are used. Molecular string enumeration can improve generative drug design, especially in low-data regimes.^42,84,85^ Recently, strategies inspired by natural language processing (such as atom masking or token deletion) have proven valuable alternatives to enumeration for de novo drug design.^86^

Early CLMs employed recurrent neural networks (RNNs).^87^ RNNs iterate over the input symbols in the sequence stepwise and compress the past information into a single memory vector called hidden state (Figure 2a). Different RNN variants exist based on the memory update rules, such as Long Short-Term Memory (LSTM)^88^ and Gated Recurrent Units (GRUs).^89^ LSTMs and GRUs were successfully used to generate syntactically valid molecules with bespoke properties.^11,16,71,82^

Transformers^90^ — an architecture that learns all pair relationships between sequence elements (Figure 2b) — were introduced to molecule design due to their transformative impact in natural language processing.^90–92^ Transformers have found widespread application in computer-assisted synthesis planning.^93^ In de novo design, they found more limited application. Recently, a comparison between transformers and RNNs have shown that the former better capture complex properties of molecules, while the latter generates more structurally diverse design libraries.^94–96^ Recently, structured state space sequence models (S4s)^20,97^ emerged as an alternative to “make the best of both worlds” by leveraging full-sequence learning (like transformers) and autoregressive generation (like RNNs). Early results suggest that S4 might enhance the diversity of design libraries compared to transformers, while better capturing complex molecular properties than both architectures.^20^

Variational autoencoders (VAEs) introduce a new component to de novo design.^98^ Rather than predicting the next symbol based on the memory, they decompose the task into two subtasks (a) learning how to encode molecules into a structured, continuous representation, and (b) reconstructing the input molecule from such learned representation.^82,98^ In a VAE (Figure 2c), an encoder network transforms an input molecule into a probability distribution over a latent space (typically modeled as a Gaussian distribution). From this latent representation, another network (decoder) learns to reconstruct molecules by sampling points from the learned distribution and mapping them back to valid input representations. The encoder and decoder can be any deep learning model, with convolutional neural networks (CNNs) and RNNs being typical choices for molecular sequences.^82,99–101^ Compared to architectures that rely solely on hidden states (e.g., LSTMs and GRUs), VAEs facilitate smoother latent space navigation, thanks to their explicit learning of latent molecule representations. These representations can be manipulated to enable tasks such as molecule optimization and property-guided design.^82^

**Figure 2:**
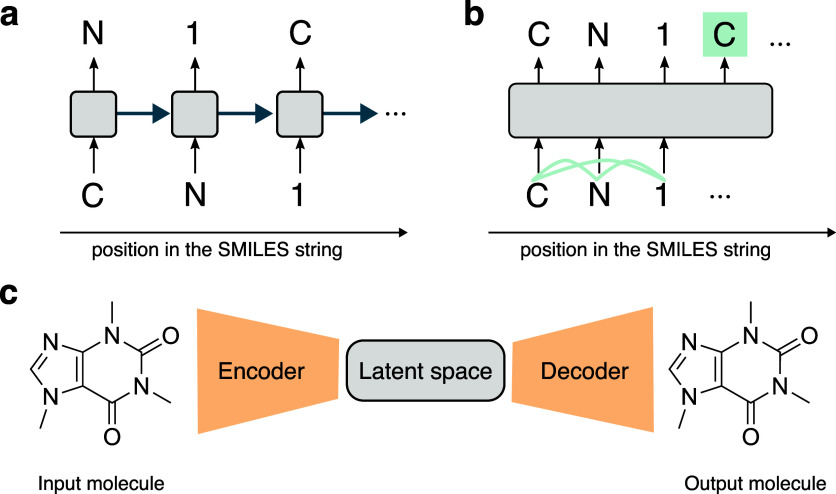
**Overview of popular deep learning architectures for de novo design.**(a) Recurrent Neural Networks, which learn to predict the next token in a SMILES string, using information on all the previous tokens. The network hidden state is updated in a recurrent way, to perform a prediction at any steps while keeping track of the preceding portions of the string. (b) Transformers, which learn all pair relationships between sequence tokens to perform a prediction. (c) Variational Autoencoder, where an encoder is trained to transform an input molecule (e.g., a graph or a string) into a fixed-dimension latent vector, and a decoder is trained to convert such vectors back into molecular representations.

Encoder-decoder architectures can also be incorporated in generative adversarial networks (GANs) for molecule design.^102–104^ Originally developed for image generation,^105^ GANs consist of (a) a generator network that learns to convert random noise into realistic instances, and (b) a discriminator network that scores the quality of the generations. These two neural networks are trained to compete with each other, with the generator aiming to produce increasingly realistic samples to “fool” the discriminator. Early applications of GANs showed promise for molecule generation, by improving drug-like properties.^104^ However, due to the challenges to train GANs—e.g., mode collapse, where the generator learns only a few modes of the input distribution^105–108^ —other score-based generators have been replacing GANs for molecular sequence design, namely diffusion^109^ models. Initially successful in generating realistic images,^109^ diffusion models learn to iteratively refine random noise into samples that resemble the input distribution. Diffusion can be applied on sequences by selecting discrete probability distributions to model the inputs, or by applying diffusion to latent molecule representation, a trend gaining popularity for sequence modeling.^110–113^ Recently developed molecular diffusion sequence models offer more controllability with more drug-like designs than initial chemical language models and form an active research direction.^114–117^

#### 2.2.2 Molecular Graphs

Like string representations, molecular graphs also gained widespread interest in de novo design using deep learning.^118–122^ Graph neural networks (GNNs) are the predominant deep learning architecture to learn from graphs.^122^ Starting from random vectors, GNNs iteratively update atom and bond representations to learn meaningful molecular representations. GNNs were first applied in combination with VAEs and GANs.^118,120,123,124^ While using GNNs as encoders is relatively straightforward, decoding graphs poses a greater challenge, as it requires reconstructing molecular structures while preserving chemical validity and similarity to the input. Calculating the reconstruction quality becomes increasingly compute-intensive as molecular size grows.^118,125^ For large molecules, using coarse-grained graphs (e.g., fragments as nodes) to shrink the graph size,^125^ and formulating graph generation as stepwise node extension can mitigate the computational burden.^118^ More recently, flow-based^126^ and diffusion^109^ models have been proposed for molecular graph generation.^50^ Flow-based models learn invertible transformations of simple probability distributions, such as Gaussian, to model the complex distribution of molecular datasets.^127–130^ Unlike VAEs and diffusion models, flow-based models can provide exact likelihood estimates of generations and provide an internal score for the designs. Diffusion models on molecular graphs learn to generate chemically valid topologies from random noise,^131–135^ Recent research on diffusion and flow-based graph models focus on improving chemical validity, scalability, and design diversity to match the performance of string generation models.^136,137^

#### 2.2.3 3D Geometry

3D approaches represent molecules as atom types and their spatial coordinates (and, optionally, bond types). Training models on atom-level 3D information allow learning from proteins and small molecules together and conditioning generations with the 3D topology of protein binding pockets. While this advantage is mostly harnessed for structure-based studies,^138^ 3D approaches have also been developed for unconditional small molecule design with diffusion.^21,58,131,139–143^ Equivariant graph neural networks (EGNNs)^144^ are a popular architecture to learn from point clouds. The equivariance provide the same, or a predictably different, output when the input molecule is rotated, translated, or reflected^144^ and provide performance gains over non-equivariant GNNs.^21^ The initial EGNNs struggled to generate valid 3D structures that satisfy bond lengths and atom stability constraints,^21^ and recent work integrated of laws of physics.^58^ and learned jointly from molecular graphs and 3D coordinates to achieve the validity level of sequence models.^55,79,145^ Using message passing transformers that respect the geometry of molecules has been recently popular, as they provide higher validity in generations.^136,145–148^ A recent study unified 3D generation and property prediction within a two-stage generation framework and improved performance in both tasks.^143^ A bottleneck for 3D generation molecule design models is the availability of datasets and benchmarks. QM9 dataset consists of computed geometric, energetic, electronic, and thermodynamic properties for 134 K molecules up to 9 heavy atoms (C, N, O, F),^6,149^ forming a narrow chemical space for drug discovery studies. GEOM-Drugs is an alternative used in 3D generation studies and contain molecules up to 91 heavy atoms and their 3D conformers.^150^ A recent improved GEOM-Drugs by reimplementing a flawed stability metric and re-evaluating the model trained upon this dataset.^151^

#### 2.2.4 Hybrid Approaches

Generating molecules in any representation has distinctive strengths, motivating approaches to combine the advantages of each. Creating a VAE with a GNNs encoder to explicitly represent the chemical structures and a CLM decoder for fast generation is one such example,^152^ resulting in higher diversity of the molecular designs. Similarly, multimodal^153^ models—which integrate multiple data types—can also be used for de novo design. Such approaches expand the information available to the models, and can even incorporate additional data sources, such as gene expressions,^154^ protein interactome,^155^ and textual information,^156^ and allow zero-shot molecule design^155^ combined with human-understandable explanations in natural language.^155,156^ Each additional modality, however, increases the architectural complexity, which can pose challenges related to computational efficiency, interpretability, and long-term scalability.

### 2.3 Focused Molecule Design with Deep Learning

- Distribution learning (Figure 3a), where a generative model is trained to learn and replicate the property distribution of a molecular dataset (e.g., physicochemical and biological properties).^10^ Distribution learning is usually achieved via transfer learning where a model is “pretrained” on a large, diverse dataset, and then “fine-tuned” on a smaller, task-specific dataset, with the same training strategy. In de novo design, a particularly useful training strategy is the next-token generation in molecular strings,^11,12^ but other strategies involve reconstructing molecular structures from corrupted inputs,^16,157,158^ or predicting missing bonds/atoms.^158–160^ An extensive body of literature using SMILES strings has shown the benefit of pretraining to learn the “syntax” of the SMILES language and global physico-chemical properties,^11,85^ and of fine-tuning to capture bioactivity, as validated in the wet-lab.^12,13,15^ Moreover, by controlling the extent of transfer learning, it is possible to indirectly navigate between the properties of the pretraining and fine-tuning molecules.^9,13,15,17,85^

- Goal-directed learning (Figure 3b), where a model is trained to optimize an external objective.^10^ This is typically implemented through reinforcement learning, in which a generative model iteratively designs molecules and receives a score (also called reward) based on an external evaluation function (e.g., docking scores^161,162^ or predicted properties^52^ and bioactivity^163^).^52,164–166^ Usually, these generative models are pretrained on large corpora of data.^52,161^ During reinforcement learning, the model updates its generation strategy based on this reward signal, “reinforcing” molecular structures that receive higher scores and discouraging those with lower scores. This iterative process helps the model refine its output toward molecules with the desired properties. Compared to distribution learning, goal-directed learning allows for a more flexible steering of molecule design toward desired regions of the chemical space, e.g., for multi-objective molecular design,^167–169^ scaffold hopping^170,171^ and hit-to-lead optimization.^172,173^

- Conditional generation (Figure 3c), where a single generative model is trained to explicitly produce molecules matching one or more desired properties. Unlike traditional goal-directed generation (which scores the de novo designs of a generation), the desirable conditions are explicitly used to train the generator in a supervised manner. This is achieved by learning a shared vector space that encodes the desired conditions (e.g., experimentally determined properties) and the corresponding molecular structures (Figure 3c) together. The desired set properties can be then used as a conditioning input to generate molecular structures that are likely to possess those properties. Conditional generation has been used to design molecules that match a desired three-dimensional shape, possess a desirable set of physico-chemical properties or substructures,^21,141,174^ or are constrained by a target protein sequence^175,176^ or gene-expression signatures.^177,178^

**Figure 3:**
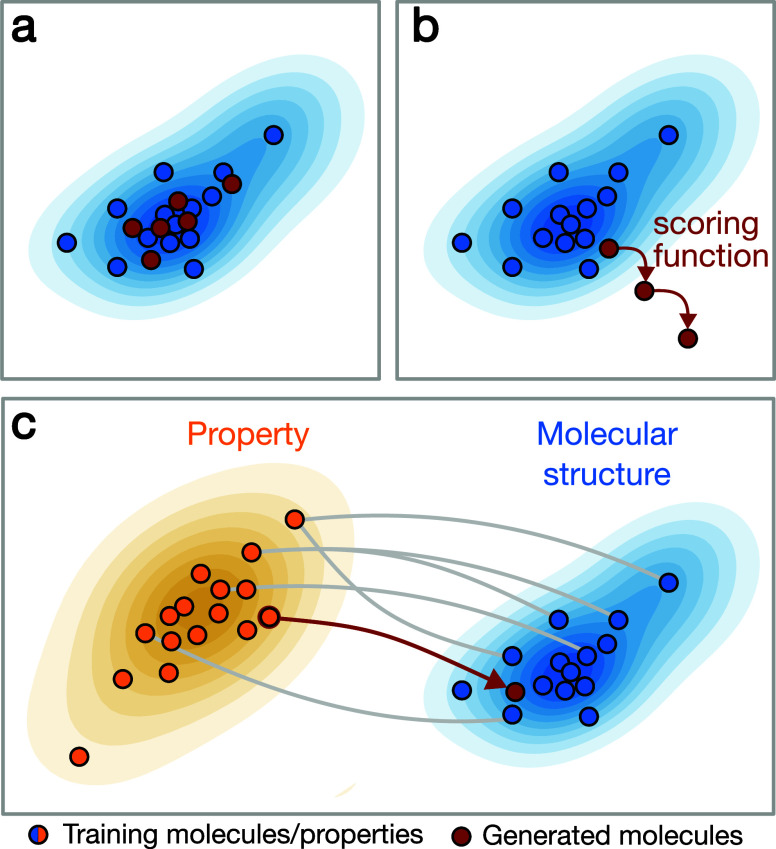
**Approaches for model training and molecular generation.** (a) Distribution learning, where models generate molecules that statistically resemble those in the training set in terms of physico-chemical and biological properties. (b) Goal-directed generation, which optimizes molecules toward a predefined objective, often using reinforcement learning, guided by a scoring function. (c) Conditional generation, where models are explicitly trained to design molecules with specified properties, by incorporating property constraints into the generation process.

Each molecular generation approach presents unique benefits and limitations, and the choice depends on a multitude of factors, such as, the availability and quality of available training data, the complexity of the target molecular properties, and specific design needs (e.g., diversity vs property optimization).

Distribution-learning methods enable end-to-end learning and molecule generation without the need for explicit scoring functions to guide the design process. Even with relatively small pretraining datasets (e.g., in the order of tens of thousands of molecules^9^) and/or fine-tuning sets (e.g., in the order of just a few dozen molecules^12,14^ ), these approaches can successfully generate molecules with desirable molecular properties, including bioactivity. Moreover, the fine-tuning process is particularly beneficial in low-data scenarios,^14,85^ where the molecules having the desired properties are too few to train a deep learning model from scratch, and the target property is resource-intensive and/or challenging to evaluate *in silico*. However, while these models are evaluated based on how well the generated molecules resemble the training set in terms of properties, they do not inherently assess the quality of individual molecules. As a result, additional scoring and filtering steps are often required to identify the most promising candidates, introducing manual intervention into otherwise “rule-free” pipelines. Finally, distribution learning might fail at property optimization if the desired characteristics are underrepresented in the training data (out-of-distribution generation).

Conversely, goal-directed strategies offer direct feedback on both individual molecules and the entire generated population through an external scoring function. Compared to distribution learning, they can, in principle, steer the generation of molecules in previously unexplored regions of the chemical space, if incorporated into a suitable scoring function. However, goal-directed generation poses several challenges,^25,26,179^ including (a) the difficulty of accurately capturing complex molecular attributes — such as bioactivity, drug-likeness, and synthetic accessibility—into a single scoring function, which might lead to molecules that do not realistically match the intended objectives; (b) the risk of models exploiting spurious correlations or unintended biases within the scoring function rather than learning meaningful structure–property relationships (a phenomenon known as “reward hacking”^180^), and (c) reduced chemical diversity due to biases imposed by the optimization process, which can even result in mode collapse.^107^ The reliability of goal-directed methods is therefore heavily dependent on the quality and diversity of the data and models used to define scoring functions. Additionally, molecules generated by goal-directed strategies may exhibit lower synthetic accessibility than those designed via distribution learning approaches.^27^ Several strategies have been explored to retain broad chemical diversity while achieving the generation objectives, such as incorporating a memory of previously produced molecules,^181^ the penalization of structurally redundant designs,^23^ or integrating transfer and reinforcement learning.^171^ Overall, whenever dealing with goal-directed generation, scoring functions play a key role in determining the properties of the de novo design. As such, scoring functions should be designed carefully, by balancing computational cost and reliability.

Finally, conditional generation has been explored less extensively in drug discovery compared to distribution-learning and goal-directed approaches. In general, however, since these models do not rely on externally computed scoring functions, they offer several potential advantages, such as (a) mitigating biases associated with predictive models (e.g., bioactivity prediction models), (b) capturing complex structure–property/activity relationships by directly associating molecular structures with desired properties within latent space, and (c) maintaining the end-to-end learning characteristics of distribution-learning methods. Despite these advantages, conditional generation has yet to be applied extensively and experimentally validated. This might be due to the fact that conditional generation is well-suited for scenarios where large molecular databases with accurate and reliable labels are used. However, such datasets are relatively uncommon in drug discovery, which might explain the limited application of conditional approaches. One potential way to overcome this limitation is to incorporate self-supervised or semisupervised approaches to enhance learning from smaller datasets. Finally, it remains uncertain whether conditional generation can effectively explore regions of chemical space beyond those represented in the training set, raising questions about its utility relative to more established generative approaches.

### 2.4 Evaluating the Quality of de Novo Designs

While generative deep learning models can rapidly produce millions of molecular designs, the evaluation of molecular quality—whether for follow-up experiments or model analysis and comparison—remains an open challenge. Despite remarkable strides in generative drug discovery, determining whether a design is “good” or “bad” remains a challenge, as it often involves balancing multiple, sometimes conflicting, objectives in a context-dependent manner. While no universally accepted guidelines exist for evaluating de novo design studies, assessment typically considers multiple factors, including chemical validity, diversity, and alignment with the intended design objectives, as outlined below.

#### 2.4.1 Chemical Validity and Non-redundancy

One of the principal requirements of a good molecular generator is its ability to generate molecules that are “chemically plausible”—in other words, molecules possessing correct valency, aromaticity and charge constraints. These aspects are usually commonly referred to as “chemical validity”. While validity only considers two-dimensional information, it has recently been extended to three-dimensional molecular generation (“validity3D”),^77^ which evaluates the conformation quality of bond lengths and valence angles. Additionally, generators should be able to produce non-redundant molecules, in terms of limiting the duplicated designs (“uniqueness”) and the overlap with the training set (“novelty”). These metrics should always be reported, as they can reveal significant issues in the model training procedure. However, validity, uniqueness and novelty are vulnerable to trivial modifications (e.g., the random insertion of a carbon atom^26^), may depend on the number of generated designs,^182^ and are easy to optimize by simple heuristic algorithms.^183^ Therefore, they should be considered as a diagnostic check rather than a conclusive evaluation metric.

#### 2.4.2 Internal Diversity of de Novo Design Libraries

Assessing the structural diversity of generated molecules is crucial to ensure that a generative model does not produce structurally redundant or overly similar compounds. High diversity is often desirable, particularly in early-stage drug discovery, as it increases the chances of identifying novel bioactive chemotypes. Evaluating and quantifying molecular diversity is not straightforward, as “similarity is in the eye of the beholder”^184^ — it depends on the chosen molecular features (molecular descriptors^185^) and similarity/diversity metrics.^186^ Often, molecular diversity is captured by computing the pairwise Tanimoto similarity^186^ (the lower, the higher the diversity) on extended-connectivity fingerprints (ECFPs),^187^ which captures the presence of shared substructures.^10,24^ Average pairwise similarities are frequently reported, at the risk of obscuring important nuances in molecular diversity. The presence of diverse molecular scaffolds^188^ is also commonly used to capture diversity.^24^ While providing a more abstract perspective on diversity compared to ECFP similarity, it can be skewed by the inclusion of scaffolds that differ only minimally.^189^ Recently, the number of circles (“#Circles”) has been introduced,^190^ which leverages sphere exclusion clustering to quantify the internal diversity of molecular datasets and measure the chemical space covered by databases and generative models. #Circles captures well the structural diversity of molecular sets^190^ and allows to better distinguish generative models with varying exploration capacities.^189^ The #Circles metric is computationally expensive, and this is why the number of unique substructures (computed via the Morgan algorithm^187^) was recently suggested as a cost-effective alternative to capture internal diversity.^182^

#### 2.4.3 Similarity to Reference Molecules

In de novo drug design tasks, generated molecules are often required to share key, desirable properties with known compounds, such as binding affinity or solubility. Consequently, their similarity to these reference compounds—often used for model training and validation—is computed as a measure of how well the designed molecules align with the desired property distributions. Like with measures of internal diversity, similarity can consider information on shared substructures, as captured by extended connectivity fingerprints (ECFPs)^187^ or Molecular ACCess System (MACCS) keys.^191^ Often, evaluations of molecular similarity are based on physicochemical descriptors, such as molecular weight, octanol–water partitioning coefficient (logP), topological polar surface area (TPSA), and number of hydrogen bond donors and acceptors.^10^ Once these descriptors are obtained for the reference molecules (e.g., training set) and the molecular designs, the degree of similarity between their distributions is computed using dedicated metrics,^10,192^ such as the Kullback–Leibler (KL) divergence^193^ or Kolmogorov–Smirnov (KS) distance^194^ (the lower the values, the more similar the distributions). Another popular measure of distance between distributions is the Fréchet ChemNet Distance (FCD).^195^ FCD is computed from the internal representations of the penultimate layer of a model for bioactivity prediction,^196^ capturing both chemical and biological information. Lower FCD values indicate greater similarity between molecular sets in terms of structure and bioactivity.^195^ An important caveat is that distribution-based similarities require a minimum number of molecules to be reliably computed,^182,195^ estimated to be around 100,000.^182^

#### 2.4.4 Predicted Molecular Suitability

Ultimately, de novo drug design aims to find molecular candidates for hit and lead discovery, possessing an array of desirable properties, such as potency, selectivity, pharmacokinetics, and safety. These properties are inherently complex and challenging to determine experimentally, due to their resource-intensive nature. This is why surrogate computational approaches of different levels are used for molecule evaluation and prioritization. Commonly used computational approaches are: (a) quantitative structure–activity relationship (QSAR) approaches,^197^ to predict biological properties such as potency, selectivity and ADMET (absorption, distribution, metabolism, excretion and toxicity) properties; (b) pharmacokinetic models,^198^ which aid in predicting bioavailability, clearance, and systemic exposure; (c) estimation of synthetic feasibility,^199–201^ at varying levels of complexity; and (d) biophysics-based approaches,^202^ such as docking and molecular dynamics. Each computational approach offers distinct advantages and limitations in molecule evaluation. QSAR models enable rapid, cost-effective predictions of biological properties but rely heavily on high-quality training data, which are often not available. At the same time, pharmacokinetic models provide physiologically relevant insights into drug disposition, yet they require accurate input parameters, which can introduce uncertainties. Synthetic feasibility estimations aid in prioritizing accessible compounds, but may bias toward well-established chemistries, potentially overlooking innovative or unconventional synthetic routes (see Section 3.3). Biophysics-based methods provide mechanistic insights into molecular interactions, with docking offering rapid but often simplified predictions of binding poses, while molecular dynamics (MD) captures conformational flexibility and stability at the cost of significantly higher computational expense. However, both approaches can suffer from scoring inaccuracies, particularly in ranking binding affinities. Despite these challenges, these computational tools have become indispensable to navigate and rationalize the large virtual libraries designed by generative deep learning.

Evaluating the quality of de novo designs remains one of the most challenging open questions in the field.^182,203^ This complexity arises from the inherently multi-objective nature of drug discovery, the unfeasibility of large-scale synthesis and biological testing, and the absence of perfect models to predict complex in vitro and in vivo properties. Nonetheless, the cheminformatics community has been particularly active in delineating guidelines and benchmarks to aid in the evaluation of the designs produced by generative deep learning algorithms (e.g., Table 2). Overall, the specific approaches adopted to evaluate a generator’s designs depend on the intended goal—whether to develop and compare generative models or to prioritize compounds for experimental validation.

**Table 2:** Overview of Existing Benchmarks for de Novo Molecular Design

Name	Evaluation	Tasks
GuacaMol^10^	General drug-like property optimization.	Distribution learning, exploration/exploitation of chemical space, single and multi-objective optimization tasks
MOSES^24^	Molecule design in general.	Distribution learning, molecular diversity, avoidance of forbidden structures.
MolOpt^227^	Sample efficiency in reinforcement learning.	Molecule optimization via 23 oracle functions.
MolScore^228^	Collection and reimplementation of Guacamol, MOSES, and MolOpt, with added evaluations.	Distribution learning, biological activity, synthetic accessibility, among others.
MolExp^230^	Chemical space exploration.	Capacity to discover dissimilar molecules that possess similar bioactivity.
QM9 ^149^	Unconditional molecule design.	3D properties of molecules up 9 heavy atoms computed via density functional theory (DFT) simulations.
GEOM-Drugs^77^	Unconditional molecule design.	Computed 3D properties of molecular conformers.
SMINA-benchmark,^231^ DOCKSTRING^232^	Docking scores.	Docking scores with various software.
GenBench3D^77^	Ligand conformational quality.^a^	Likelihood of bond lengths and valence angles based on reference values from the Cambridge Structural Database (CSD).
CBGBench^233^	Quality of structure-based de novo design.^a^	Evaluation of structure-based de novo design across four generation tasks.

a only for 3D generation approaches.

When comparing generative models or developing new ones, large-scale assessment is recommended, typically involving at least 100,000 molecular designs.^182^ In this context, evaluating chemical validity and non-redundancy are necessary, but not sufficient, to monitor the correct learning of chemical information. Furthermore, the analysis of similarity and diversity patterns (e.g., number of circles or substructures, descriptor similarity and FCD) are required to provide insights into a model’s potential and limitations. Practitioners should ensure that the same number of generated designs should be used across evaluations, to prevent unintended confounding factors that could bias model comparison.^182^

For applications aiming to bring generative models closer to experimental validation, a more refined evaluation strategy might be required. While similarity vs. diversity analysis remains relevant for optimizing the exploration-exploitation trade-off, additional layers of molecular assessment might come into play. Early-stage filters, such as simple synthetic accessibility estimations, help narrow down the chemical space before applying more sophisticated and computationally demanding retrosynthesis predictions. Similarly, biophysics-based evaluations may start with docking for rapid binding predictions, followed by more resource-intensive molecular dynamics simulations to refine binding mode stability and interaction profiles.

To date, the effectiveness of de novo design evaluation, especially for prospective applications, remains dependent on the expertise and viewpoint of the researchers analyzing the results, highlighting the need for a strong synergy between machine learning specialists and medicinal chemists to ensure that computationally generated designs align with real-world drug discovery objectives.

## 3 With Complex Objectives Come Complex Responsibilities

### 3.1 The Similarity-Diversity Paradox

Designing new drug candidates faces a persistent challenge: balancing structural diversity with molecular similarity to known molecules. On one hand, similarity to known bioactive compounds increases the likelihood of identifying viable drug candidates, as structurally related molecules often share biological activity.^204^ On the other hand, excessive similarity can restrict chemical innovation and the likelihood of charting novel chemical space. This not only hampers the discovery of molecules with improved therapeutic properties—such as enhanced potency, selectivity, or safety—but also raises concerns regarding patentability of compounds that closely resemble existing drugs. In drug discovery, innovation beyond “me-too” compounds^205^ requires striking a balance between leveraging known bioactivity and exploring sufficiently distinct chemical matter to uncover new mechanisms of action and therapeutics. We term this tension as the “similarity-diversity paradox”, to underscore the difficulty in simultaneously achieving the two.

How to balance similarity and diversity has been a long-standing question in the cheminformatics and drug discovery communities.^206,207^ This paradox can be mitigated by aiming to minimize substructure and scaffold similarity, while preserving three-dimensional information. In the context of protein–ligand binding, in fact, bioactivity is primarily driven by three-dimensional shape and electrostatics complementarity,^208^ along with other molecular recognition factors.^209^ Based on this concept, several generative approaches have incorporated shape and/or electrostatic complementarity in the design process, e.g., by conditioning the generation using pharmacophore or three-dimensional pocket information,^210–213^ or via ad-hoc scoring for reinforcement learning.^171,214,215^ These approaches could allow to preserve core features for binding (i.e., similarity to pharmacophore and/or 3D shape and electrostatics) while allowing to explore new regions in the chemical space in terms of scaffolds and substructures (ensuring “two-dimensional” diversity). Another strategy to increase diversity is by drawing inspiration from natural products.^216,217^ Natural products have been a rich source for medicinal chemists due to their structural complexity and diverse scaffolds, which often translate into a wide range of biological activities.^218–220^ Recognizing this, several studies have employed deep learning techniques to generate natural product-inspired compounds via transfer learning^13,17,44^ or fragment-based generation.^221^ These studies show that incorporating natural-product-derived information allows to expand the chemical space accessible for drug discovery while preserving desirable properties such as bioactivity.

Exploring previously uncharted regions of chemical space is nevertheless not easy. In fact, most available molecule evaluation tools—such as docking algorithms, QSAR models and ADMET prediction tools—might struggle with highly novel scaffolds.^222–224^ These tools often rely on statistical correlations derived from existing datasets, meaning that molecules with unprecedented cores or non-traditional structural features may fall outside their applicability domain. For instance, docking algorithms typically assume that ligands share similarities with known binders, leading to inaccurate scoring for highly distinct chemotypes.^223^ Similarly, machine learning-based bioactivity predictors are known to provide unreliable predictions when applied to molecules that are too different from the molecules used for training.^224,225^ Finally, synthetic accessibility scores often penalize novel molecular structures, and might not consider current or future advancements in organic synthesis.

Navigating the similarity-diversity paradox with the current technologies ultimately requires combining several techniques. De novo designs occupying the “familiar”^225^ chemical space can be scored with the available data-driven tools such as docking approaches and predictive models, and synthesis routes can be devised for prospective studies. For diverse designs outside the applicability domain, physics-based molecular dynamics simulations of increasing computational complexities can be run. Initial short or coarse-grained simulations can help preselect a smaller pool of de novo designs. For these selected candidates, performing more extensive and accurate simulations (potentially with multiple replicates) might help the refinement of the compound selection. Ultimately, overcoming the similarity-diversity paradox will be only possible by combining efforts from multiple disciplines, such as improving generalizability in molecular machine learning, and finding synergies, complementarities and overlaps between physics-based approaches and data-driven predictions.

### 3.2 Benefits and Limitations of Molecular Benchmarks for de Novo Design

Evaluating generative models poses additional challenges than evaluating predictive models (e.g., to predict bioactivity or toxicity). While predictive models can be evaluated via held-out molecules that were previously tested,^226^ de novo designs are, by definition, molecules that have not been previously tested for their properties. This makes it difficult to evaluate different model architectures, choose which model should be used for prospective studies, and identify learning gaps. To overcome this limitation, in the past few years, several notable benchmarking efforts,^10,24,227,228^ such as GuacaMol^10^ and MOSES,^24^ have strived to provide standardized datasets and metrics, to evaluate and compare generative models (Table 2).

GuacaMol^10^ and MOSES^24^ are to date the most well-known benchmarking platforms. They include datasets for testing, along with benchmarking metrics for comparing generative models—with GuacaMol comprising both distribution-learning and goal-directed models, while MOSES focusing on distribution-learning models. These benchmarks have enabled researchers to compare diverse approaches systematically, facilitating rapid progress in algorithmic development and model optimization. By offering well-defined tasks (e.g., optimizing molecular properties, generating diverse compounds, or mimicking training distributions), these benchmarks have become indispensable tools for assessing the performance and utility of generative algorithms in chemistry. While molecular benchmarks are undeniably valuable, there are inherent limitations and potential pitfalls associated with their use. Prominent among these is the risk of overfitting models to benchmark-specific tasks, metrics, or datasets. Such overoptimization can lead to a “tunnel vision”, where models excel at predefined tasks but fail to generalize to real-world drug design challenges. In this context, excessive reliance on benchmarks has been suggested to stifle creativity, as researchers prioritize improving benchmark scores over addressing broader, more impactful scientific questions.^229^ Moreover, molecular benchmarks often simplify the complexity of drug discovery. Real-world drug design involves multifaceted objectives, iterative processes, and the integration of experimental validation—aspects that are not easily captured in benchmark tasks. The reliance on computational metrics, such as similarity or property optimization, may inadvertently bias research toward generating compounds that are easy to evaluate computationally but less relevant for practical applications.

To address these challenges, the field must strike a balance between leveraging the advantages of benchmarks and fostering innovative, application-driven research. This includes testing and benchmarking approaches on more than one benchmark, continue the development of progressively more realistic evaluation metrics and datasets, and ultimately, incorporate experimental feedback, whenever possible. By acknowledging both the benefits and limitations of molecular benchmarks, we wish for the community to harness the potential while remaining vigilant against their unintended consequences.

### 3.3 Navigating Feasibility in Molecular Generation

While generative deep learning models allow to produce previously unseen molecules, difficulties arise in addressing synthetic feasibility.^27^ Defining and incorporating synthesizability is a challenging task, as it may depend on subtle structural variations, reaction selectivity, and the commercial availability of building blocks essential to chemical reactions.^28^ Moreover, several research has shown that the synthesizability of molecules proposed by deep learning may be limited, depending on the chosen approach.^27^

A wealth of research has focused on assessing the synthetic feasibility of molecules. Commonly used strategies generally assess one of the following aspects (Table 3):^234^

- *Synthetic complexity scores*, identify those molecular characteristics that could challenge the synthesis. Metrics such as the synthetic accessibility score (SASscore)^199^ estimate synthetic complexity based on the presence of predefined molecular motifs, such as the number of bridged and spiro systems, stereocenters, or macrocycles. Other complexity metrics account for functional group distribution, molecular size, and ring system strain.^235^ More recently, scores of synthetic complexity based on deep learning have been introduced, for instance, based on the number of estimated reaction steps required.^200^
- *Predicted retrosynthesis routes.*Determining retrosynthesis routes involves decomposing a target molecule into building blocks that are either commercially available or easy to synthesize.^236^ Computationally, retrosynthesis planning can be approached by using either rule-based methods or machine learning. Rule-based approaches^237,238^ identify functional groups and reaction motifs, then apply a library of predefined reaction templates to suggest potential precursors. Machine learning approaches are used to learn retrosynthetic feasibility directly from large databases of annotated reactions. This can be performed via (a) template-based retrosynthesis, where the model selects reaction templates from training data,^239,240^ or (b) template-free retrosynthesis, where sequence-to-sequence learning or graph-based transformations are applied to generate precursors directly, without predefined templates.^241–243^

**Table 3:** Selected Approaches to Assess and Incorporate Synthetic Feasibility in Molecular Generation. The approaches are divided by type, and subtypes, along with the selected examples.

Type	Description	Examples
Synthetic complexity scores	Penalizes complex structures, such as fused rings or stereocenters.	Synthetic Accessibility Score (SASscore)^199^
	Determines a synthesis tree to choose the most favorable route.	RASA^254^
	Based on deep learning and the estimated number of reaction steps.	SCscore,^200^ FSscore^255^
Retrosynthetic Analysis of designed compounds	Rule-based planning using predefined reaction rules.	CAS Scifinder,^237^ Chematica^238^
	Template-free models predicting reactants from the product (or vice versa) using deep learning.	Wan et al.,^241^ Tetko et al.,^242^ Yao et al.^243^
	Template-based deep learning approaches based on learned transformations.	AiZynthFinder,^256^ IBM RXN,^257^ Synthia^256^
Synthesizability by design	Systematic generation of possible candidates with a predefined reaction rules.	RENATE,^258^ DINGOS^259^
	Constrained molecular generation to favor synthesizable compounds, often via reinforcement learning.	SynFormer,^247^ TANGO,^249^ ClickGen,^250^ REACTOR,^165^ Guo and Schwaller^245^

Each strategy has distinct advantages and disadvantages. Synthetic complexity scores provide a rapid assessment of synthetic feasibility, which makes them particularly suited to evaluate large molecular libraries, or to be included in reinforcement learning pipelines. However, synthetic complexity scores that rely on predefined heuristics are static as they may not include new synthetic methodologies and cannot easily be updated based on new reaction data. Furthermore, these strategies condense vast information into a single score, which can obscure important nuances, such as whether a high complexity score is due to challenging stereochemical features, strained ring systems, or uncommon functional groups. As a result, these scores may sometimes penalize molecules that are accessible via modern synthetic routes.

In contrast, machine learning-based approaches offer a dynamic and data-driven alternative to static complexity scores. By learning from large reaction databases, they can capture nuanced relationships between molecular structures and their synthetic feasibility while adapting to new reaction data. Lastly, retrosynthesis-based models further provide explicit synthetic routes, with template-based methods using established transformations and template-free approaches offering greater flexibility for novel chemistry. However, these models are limited by data bias and reaction coverage.

For this reason, the choice of feasibility assessment method depends on the application. Complexity scores remain valuable for rapid screening of large molecular libraries, and computational pipelines where computational efficiency is crucial. Meanwhile, machine learning-based retrosynthesis models provide more practical feedback for experimental applications by suggesting synthesis routes, but they require careful curation of reaction data to ensure reliability.^244^ While synthetic feasibility is typically assessed after molecule generation, approaches that generate molecules while considering the synthesizability constraints were also developed. They are divided into following groups:^245^

- Enumeration-based methods,^245^ which refers to the systematic generation of (all) possible candidate of building blocks molecules by following a set of predefined reaction rules. This method explores the synthetic accessible space, often using reaction vectors for the reaction rules.^245,246^

- Synthesizability-constrained molecular generation,^245^ which include reaction templates, or reactivity predictions to generate experimental accessible molecules and their pathways.^247,248^ Another promising approach is to integrate synthetic accessibility as an optimization objective within reinforcement learning, to steer the generation toward drug-like synthetically accessible molecules.^165,249,250^ Other approaches have used autoencoders for molecule optimization that considers both desirable molecular properties and elements of synthetic accessibility,^28,251–253^ and optimized the score of oracle retrosynthesis prediction models.^245^

Last, estimating synthesizability adds to the challenges of designing structurally diverse (and out of distribution) molecules, while remaining synthetically feasible. In these cases, the designs might not only fall out of distribution of the approaches used to assess their physico-chemical and biological properties, but also of the approaches used to estimate their synthesizability. To bridge this gap, recent research has addressed the generalizability of synthesis prediction tools when applied to newly reported patents or reactions.^260^ Advancing synthesis estimation models in parallel with molecule generation ones is expected to be a key strategy to allow charting the chemical universe in an efficient manner.

### 3.4 The Experimental Validation Dilemma

Unlike bioactivity or molecular property prediction—where models are usually evaluated on well-defined test sets—molecular generation aims to propose novel, previously unseen molecules, whose properties are unknown. Moreover, generative approaches tend to show low “molecular rediscovery” rates,^192,261^ making it difficult to use existing, held-out molecules for the evaluation. These aspects make direct evaluation of generative approaches difficult, as there is no ground truth to compare against. Compounding this challenge, existing tools for assessing de novo designs—such as similarity metrics, bioactivity prediction, or heuristic scores—are inherently imprecise and/or only partially capture complex design objectives.

Arguably, the only definitive “proof-of-the pudding” of bioactivity is experimental validation. However, experimental testing is expensive and time-consuming, meaning that only a small fraction of generated molecules can be synthesized and evaluated. Hence, this process faces two major bottlenecks:

- *Selection of molecules to make and test.* With thousands of generated designs scoring similarly on computational metrics, choosing which ones to validate experimentally is challenging. Many of these scores contain inherent noise, and small variations in ranking may not correspond to meaningful differences in experimental properties. At the same time, errors in the scoring functions (e.g., for out-of-distribution molecules^225^) might rule out suitable molecules. Ensuring that promising candidates are not overlooked requires robust selection strategies—yet, defining optimal criteria for this process remains an open challenge.
- *Sufficiency of experimental validation.* In machine learning, test sets used to validate predictive models, typically contain a substantial number of molecules to provide statistically robust evaluations (e.g., in the order of hundreds to thousands of compounds^262^ ,^263^ ). In de novo design, however, synthesizing and testing even a small fraction of generated molecules is prohibitively expensive. This raises a fundamental challenge: *how many designs are sufficient to reliably measure the effectiveness of (and compare) generative deep learning models?* To date, balancing practical feasibility with robust evaluation remains an unresolved issue.

In addition to cost and time constraints, experimental validation of generative deep learning approaches demands expertise across multiple disciplines, including synthetic and medicinal chemistry, computational modeling, assay development, and data analysis. This interdisciplinary challenge likely contributes to the relatively scarce experimental validation of generative models (Table 4), especially when compared to the vast number of proposed approaches. This might also be the reason why the field tends to validate new methods predominantly on well-explored targets, like kinases and nuclear receptors (Table 4). Finally, the validation cost and complexity escalate as the targeted property becomes more clinically relevant, requiring more advanced assays, *in vivo* studies, or even early stage pharmacokinetics and toxicity evaluations. These challenges underscore the difficulty of translating de novo molecular design from computational predictions to real-world applications.

**Table 4:** Experimentally Validated Studies Involving Generative Deep Learning for Hit Design (Updated May 2025).^c^

Superfamily	Targ**et**	Model type	Model(s)^a^	Potency range^b^	Reference
Kinases	Janus Kinase 3 (JAK3)	CLM (SMILES)	Entangled Conditional Adversarial Autoencoder	IC_50_ = 6.73 μM	Polykovskiy et al. 2018 ^267^
Kinases	Discoidin Domain Receptor 1 (DDR1)	CLM (SMILES)	VAE	IC_50_: 0.010–0.278 μM	Zhavoronkov et al. 2019 ^268^
Kinases	Discoidin Domain Receptor 1 (DDR1)	CLM (SMILES)	LSTM	IC_50_: 0.092–2.239 μM	Yoshimori et al. 2020 ^269^
Kinases	FMS-like tyrosine kinase 3 (FLT-3)	CLM (SMILES)	LSTM	IC_50_ = 1.98 μM	Jang et al. 2022 ^270^
Kinases	Epidermal growth factor receptor (EGFR)	CLM (SMILES)	RNN	pIC_50_: 5.9–7.4	Korshunova et al. 2022 ^271^
Kinases	Receptor-interacting protein kinase 1 (RIPK1)	CLM (SMILES)	Conditional LSTM	IC_50_: 0.035–0.463 μM	Li et al. 2022 ^272^
Kinases	Phosphoinositide 3-kinase gamma (PI3Kγ)	CLM (SMILES)	LSTM	K_d_: 0.013–0.29 μM	Moret et al. 2023 ^16^
Kinases	Cyclin-dependent Kinases (CDK1/2)	CLM (SMILES)	Fragment-Based VAE	IC_50_: 0.0015–8 μM	Yu et al. 2023 ^273^
Kinases	Cyclin-dependent kinase 8 (CDK8)	Mix	Ensemble of generative models^c^	IC_50_: 0.0004 μM	Li et al. 2023 ^274^
Kinases	Salt-inducible kinase 2 (SIK2)	Mix	Ensemble of generative models^c^	IC_50_: 0.14–9.8 μM	Zu et al. 2023 ^275^
Kinases	Cyclin-dependent Kinase 2 (CDK2)	Geometry-based (3D atom positioning)	Diffusion	IC_50_ < 0.001 μM	Huang et al. 2024 ^22^
Kinases	Cyclin-dependent kinase 20 (CDK20)	Mix	Ensemble of generative models^c^	K_d_: 0.034–9.2 μM	Ren et al. 2024 ^276^
Kinases	Polo-like kinase 1 (PLK1)	CLM (SMILES)	Transformer conditioned on pharmacophores^b^	IC_50_: 0.00510.037 μM	Xie et al. 2025 ^211^
Kinase	TRAF2- and NCK-interacting kinase (TNIK)	Mix	Ensemble of generative models^c^	IC_50_: 0.0048–0.0078 μM	Ren et al. 2025 ^277^
Nuclear receptors	Peroxisome Proliferator Activated Receptors (PPAR); Retinoid X Receptors (RXR)	CLM (SMILES)	LSTM	EC_50_: 0.06–10.1 μM	Merk et al. 2018 ^12^
Nuclear receptors	Retinoid X Receptors (RXR)	CLM (SMILES)	LSTM	EC_50_: 16.9–15.7 μM	Merk et al. 2018 ^13^
Nuclear receptors	Liver X receptor α (LXRα)	CLM (SMILES)	LSTM	EC_50_: 0.183–1.31 μM	Grisoni et al. 2021 ^15^
Nuclear receptors	Retinoic acid receptor-related orphan receptor γ (RORγ)	CLM (SMILES)	LSTM	EC_50_:0.68–4.6 μM	Moret et al. 2021 ^17^
Nuclear receptors	Orphan nuclear receptor related 1 (Nurr1)	CLM (SMILES)	LSTM	EC_50_: 0.04–2.1 μM	Ballarotto et al. 2023 ^14^
Nuclear receptors	Proliferator-Activated Receptors (PPAR)	CLM (SMILES) with graph conditioning	GTNN + LSTM^c^	EC_50_: 0.24–2.3 μM	Atz et al. 2024 ^155^
Nuclear receptors and enzymes	Proliferator-Activated Receptor (PPARδ), and soluble epoxide hydrolase (sEH)	CLM (SMILES)	LSTM	EC_50_:0.009–0.022 μM; IC_50_: 0.005–0.097 μM	Isigkeit et al. 2024 ^278^
Other	K-opioid receptor (KOR)	CLM (SMILES)	VAE	K_i_: 6.46–7.59 μM	Salas-Estrada et al. 2023 ^279^
Other	Tuberculosis ClpP	CLM (SMILES)	GPT-like^c^	IC_50_: 1.88–35.2 μM	Wu et al. 2024 ^280^
Other	Histone acetyltransferase 1 (HAT1) and YTH domain-containing protein 1 (YTHDC1)	Geometry-based (3D atom and bond positioning)	Autoregressive flow^c^	IC_50_ (HAT1) = 72.36 μM; IC_50_ (YTHDC1) = 32.6 μM	Jiang et al. 2024 ^281^
Other	Bacteria (A. baumannii)	Fragment-based	MCTS	MIC ≤8 μg mL^–1^	Swanson et al. 2024 ^282^
Other	Prolyl hydroxylase domain (PHD) enzymes	Mix	Ensemble of generative models^c^	IC_50_ = 0.004 μM	Xu et al. 2024 ^283^
Other	Kirsten rat sarcoma virus (KRAS)	CLM (SMILES)	LSTM and QCBM	EC_50_: 0.9–24.6 μM	Ghazi Vakili et al. 2025 ^284^
Other	Monoglyceride lipase (MGLL)	Graphs	GTNN	pIC_50_: 3.66–5.99	Hassen et al. 2025 ^285^

a GPT = generative pre-trained transformer; GTNN = graph transformer neural network; LSTM = long short-term memory; MCTS = monte carlo tree search; QCBM = quantum circuit Born machines; RNN = recurrent neural network; VAE = variational autoencoder.

b EC_50_: = half maximal effective concentration; IC_50_: = half maximal inhibitory concentration; K_d_ = dissociation constant; K_i_ = inhibition constant; MIC = minimum inhibitory concentration; pIC_50_ = negative logarithm of IC_50_.

c Only studies reporting fully de novo designed molecules, without subsequent structural modifications, are included. All models are ligand-based, unless specified.

The integration of automated synthesis and self-driving laboratories offers a promising solution to such “experimental validation dilemma”.^15,264^ By combining robotic synthesis, high-throughput screening and deep-learning driven molecule design and synthesis planning, these platforms can accelerate experimental validation while mitigating the bottlenecks of manual experimentation. Automated synthesis not only enables a more systematic assessment of the capabilities of generative drug discovery approaches,^15^ but also enhance the efficiency of design-make-test-analyze cycles. Incorporating adaptive learning strategies (e.g., active learning^265,266^ ) could further refine molecular generation strategies, guiding the exploration of the chemical space with greater precision and versatility.

## 4 Future Directions and Opportunities

Generative deep learning has advanced the opportunities to design molecules on-demand in search for drug hit and lead candidates. The field is experiencing rapid advances, offering both exciting prospects and notable challenges.

One of the biggest challenges remains the evaluation of design quality. In complex design tasks, such as the design of bioactive molecules, accurate and scalable quality metrics are lacking. Fast evaluation metrics, e.g., quantitative estimate of drug-likeness^286^ and QSAR model predictions, can misguide molecule selection and sacrifice prediction accuracy.^262,287–289^ On the other hand, relying on accurate but time-consuming simulations is practically unfeasible on large libraries, and hence it inevitably constrains the breadth of the analysis. We see accelerating these simulations with deep learning as a promising research direction,^290–292^ which could remove a major obstacle to realizing the full potential of generative deep learning. Until speed and accuracy are effectively combined, new research should report multiple and multifaceted evaluation metrics for large design libraries to better reflect design quality.^25,26,182^

A direct consequence of the challenges in design evaluation is increased risk of testing the proposed de novo designs in the lab. While computational scoring functions (e.g., docking and QSAR models) can prioritize de novo designs,^211,293^ they are reliable only within their respective applicability domains, leading to filtering out structurally novel designs.^294^ Hence, training models with better out-of-distribution capabilities can extend these limits and represents a promising research direction. We expect the integration of predictive approaches with higher generalization capabilities into reinforcement learning as a promising direction to enable fast chemical space exploration in search for structurally novel molecular entities.

Another fundamental challenge for de novo design is data scarcity.^295,296^ Even “large” bioactivity datasets usually contain only a few thousand molecules^297,298^ which constitutes a bottleneck for deep learning. Transfer learning helps mitigate this problem by leveraging unlabeled datasets, however, current approaches also face issues such as catastrophic forgetting^299^ and mode collapse,^107^ resulting in reduced design diversity. Designing new training strategies has attracted increasing attention from the research community. In-context learning for molecules^300–302^ and test-time scaling of generations^303^ are two promising examples; further research is needed to validate their applicability. We also view inactive molecules as an invaluable resource for low-data drug discovery research, since inactive molecules are typically more abundant than active ones.

It is impossible to ignore the recent rise of large language models (LLMs) and their potential to accelerate molecular design. While open questions remain regarding their chemical representation capabilities,^304–308^ these models offer great ability to integrate diverse sources of information, from textual descriptions of chemical reactions to structured datasets, potentially leading to rapid and/or more informed molecular generation and design evaluation.^309–312^ However, despite their promise, LLMs have yet to fully permeate drug discovery, and their role in addressing key challenges such as generalization, zero-shot learning, and structure-based design remains an open question.

We currently see two parallel but increasingly intertwined movements. The deep learning community continues to push the boundaries of generative modeling, developing more powerful architectures to explore chemical space.^20,131,300,313^ Meanwhile, the drug discovery community is working to integrate key factors (e.g., chemistry, bioactivity, synthesizability) into the evaluation of generative models, to ensure their relevance for therapeutic applications.^261,314,315^ Historically, these efforts have often progressed in isolation, but they are now converging as both fields recognize the need for models that are not only innovative but also grounded in medicinal chemistry and pharmacology. Bridging this gap will require strong interdisciplinary collaborations between these communities. Moreover, advances in model interpretability, evaluation frameworks that better reflect real-world constraints, and tighter integration between computational design and experimental validation, will help close such gap. As these two domains continue to evolve, the true measure of success will not be in generating molecules alone, but in producing candidates that can withstand the demands of drug development and make a tangible impact. Herein, the importance of not only publishing success stories, but also negative experimental results are key, as it helps researchers understand better where research pipelines involving computational methods need to be adjusted.
